# Candidate gene mapping identifies genomic variations in the fire blight susceptibility genes *HIPM* and *DIPM* across the *Malus* germplasm

**DOI:** 10.1038/s41598-020-73284-w

**Published:** 2020-10-01

**Authors:** Richard Tegtmeier, Valerio Pompili, Jugpreet Singh, Diego Micheletti, Katchen Julliany Pereira Silva, Mickael Malnoy, Awais Khan

**Affiliations:** 1 Plant Pathology and Plant-Microbe Biology Section, Cornell University, Geneva, NY 14456 USA; 2 Research and Innovation Centre, Fondazione Edmund Mach, San Michele all’Adige, Italy

**Keywords:** Genetics, Agricultural genetics, Genetic linkage study, Genetic markers, Genotype, Plant breeding, Plant genetics, Quantitative trait, Plant breeding

## Abstract

Development of apple (*Malus domestica*) cultivars resistant to fire blight, a devastating bacterial disease caused by *Erwinia amylovora,* is a priority for apple breeding programs. Towards this goal, the inactivation of members of the *HIPM* and *DIPM* gene families with a role in fire blight susceptibility (*S* genes) can help achieve sustainable tolerance. We have investigated the genomic diversity of *HIPM* and *DIPM* genes in *Malus* germplasm collections and used a candidate gene-based association mapping approach to identify SNPs (single nucleotide polymorphisms) with significant associations to fire blight susceptibility. A total of 87 unique SNP variants were identified in *HIPM* and *DIPM* genes across 93 *Malus* accessions. Thirty SNPs showed significant associations (*p* < 0.05) with fire blight susceptibility traits, while two of these SNPs showed highly significant (*p* < 0.001) associations across two different years. This research has provided knowledge about genetic diversity in fire blight *S* genes in diverse apple accessions and identified candidate *HIPM* and *DIPM* alleles that could be used to develop apple cultivars with decreased fire blight susceptibility via marker-assisted breeding or biotechnological approaches.

## Introduction

Breeding perennial trees and woody crops is a challenge due to their long juvenile periods, slow growth rates, and high heterozygosity. A combination of next-generation sequencing and marker-assisted selection (MAS) can overcome many of these challenges and reduce the time needed for each selection cycle^1–6^. Given the economic impact, fruit quality and, more recently, disease resistance have been the major focus for apple breeders. The introgression of disease resistance into commercially preferred backgrounds is often problematic, given that the majority of known major effect resistance loci exist within wild species^7^. Wild *Malus* species often have poor fruit quality and it can take up to 25–30 years to fully recover fruit quality and eliminate linkage drag of undesirable alleles in a *Malus domestica* background^8^. Self-incompatibility and high heterozygosity also make breeding outside of *M. domestica* not feasible in many cases^9^. Thus, within this context, developing sequence-based markers and using them in targeted MAS can greatly reduce the length of breeding schemes.

The molecular markers linked with traits can be identified using bi-parental QTL (Quantitative Trait Loci) mapping, genome-wide (GWAS) or candidate gene-based association mapping approaches. The GWAS approach is based on the use of several thousand markers evenly spaced across the whole genome that can be implemented to identify QTLs. Candidate gene-based association mapping uses molecular markers from genes with known trait-associated functions from literature to identify the genetic variants that most affect the phenotype. In *Malus*, this approach has been used to define impacts on the depolymerization of pectin and changes in the resulting fruit texture of variants in the *Md-PG1* gene identified by resequencing^10^. These gene-based association studies highlight the importance of identifying specific markers for trait-specific MAS.

Fire blight, caused by the gram-negative bacterium *Erwinia amylovora*, has a devastating economic impact on apple, pear, and quince production worldwide^6,7,11,12^. The limited resistance to fire blight in modern commercial apple cultivars has led to increased reliance on antibiotics, growth inhibitors, and crop insurance claims^13^. In different *Malus* species, several major and minor effect fire blight resistance QTLs have been identified. For example, major resistance QTLs were found in *M. robusta, M. floribunda, M. ‘Evereste’,* and *M. fusca* on linkage groups 3, 12, 12, and 10, respectively^14–16^. The challenges of conventional apple breeding make these QTLs most useful for marker-assisted breeding or genome editing. Studies have also identified minor to moderate-effect fire blight resistance loci within *M. domestica*. For example, QTLs have been identified on ‘Fiesta’, ‘Prima’, and ‘Discovery’ on linkage group 7, and in ‘coop16’ and ‘coop17’ on linkage groups 2, 6, and 15, respectively^17–19^. Due to the rapid rate of evolution of *E. amylovora,* these resistance QTLs, particularly major effect QTLs, can be overcome rather quickly^20,21^*.* For example, a non-synonymous SNP in the *AvrRpt2EA* effector gene could overcome major fire blight resistance gene *FB_MR5*, derived from an accession of *Malus robusta*^20,21^.

Susceptibility (*S*) genes can provide a broad-spectrum and more durable alternative to narrow-spectrum and pathogen-specific resistance genes to develop cultivars with reduced disease susceptibility. Resistance genes typically recognize specific pathogen-encoded avirulence (Avr) proteins to induce the resistance reponse. Whereas, *S* genes facilitate host recognition and compatibility by encoding negative regulators of immune signaling, as well as providing for successful pathogen penetration, establishment and proliferation, therefore, reducing their activity can lead to a more resistant plant^22^. The use of susceptibility genes in breeding biotic stressors has been relatively limited. *X5*, *Xa13*, and *eIF4G* in rice as well as *eIF4E* in barley, pepper, melon, pea, and lettuce are a few S genes that have been targets of breeding efforts^23–32^. Susceptibility genes are well suited for genome editing, as targeted knockouts via CRISPR/Cas9 or TALENs can subtract the genetic contribution of *S* genes, decreasing the disease susceptibility of the plant. A knockout of the grapefruit *S* gene *CsLOB *via CRISPR/Cas9 resulted in reduced infection by the citrus canker pathogen (*Xanthomonas citri* ssp.) compared to the wild type (Duncan grapefruit; *Citrus* × *paradisi*)^33^. For *Malus*, two susceptibility genes, *HIPM* and *DIPMs* (*DIPM1-4*) have been identified as key regulators of establishment and proliferation of *E. amylovora*^34,35^. HIPM (HrpN-interacting protein from *Malus*) interacts with the primary class of effector proteins (HrpN) in *E. amylovora* that initiate infection, the production of reactive oxygen species, and the induction of hypersensitive response in the host^36–38^. HIPM is a transmembrane protein of 60 amino acids and was demonstrated to be expressed constitutively in apple tissues, particularly in flowers, the most critical tissue for *E. amylovora* infection^36^. The reduction in expression via RNAi of *HIPM* in the cultivar ‘Galaxy’ resulted in decreased fire blight susceptibility in the transgenic plants^35,38^. Similarly, DIPMs (DspA/E-interacting proteins from *Malus*) are apple proteins encoded by a family of four genes (*DIPM1-4*, with length of 666, 676, 665 and 682 amino acids, respectively) and are a close match to transmembrane leucine-rich repeat receptor-like kinase sequences in other organisms^39^. *DIPMs* are expressed in young shoots and their transcript level increases upon *E. amylovora* infection, except for *DIPM1*^39^. Moreover, using yeast two-hybrid and pull-down assays, Meng and colleagues^39^ demonstrated that the kinase domains of DIPM1, 2, and 4 interact physically and specifically with the *E. amylovora* effector protein DspA/E. On the contrary, DIPM3 is not subjected to this interaction. Within this framework, the same authors preliminarily concluded that DIPMs may act as susceptibility factors during the apple-*E. amylovora* interaction. Recently, it was shown that the inactivation of *MdDIPMs *via RNAi or CRISPR/Cas9 in the commercial cultivars ‘Gala’ and ‘Golden Delicious’ altered the disease phenotypes of the edited plants and reduced their susceptibility levels to *E. amylovora* strain Ea273^34,40^, thus effectively confirming the involvement of *DIPMs* in apple susceptibility.

This study identifies putative non-functional alleles of *HIPM* and *DIPM* genes from publicly available apple genomes, investigates the genomic diversity of *HIPM* and *DIPM* genes in *Malus* germplasm collections, evaluates a diverse set of *Malus* accessions for fire blight resistance in the greenhouse, and uses a candidate gene-based association mapping approach to identify SNPs (Single nucleotide polymorphisms) in these genes showing significant association with fire blight susceptibility phenotypes.

## Materials and methods

### Plant material

A total of 238 apple accessions, from four different sources, were included in this study (Supplementary Table S1). Collection 1 had a total of 145 *Malus* accessions with whole-genome resequencing data. These are from three different sources, the majority from *M. domestica*, *M. sieversii,* and *M. sylvestris* that were previously sequenced^41^, 67 accessions that were sequenced separately^42^, and eight further accessions of *M. sieversii* and *M. sylvestris* sequenced in this study (unpublished data). Collection 2 had 93 accessions from 26 *Malus* species that were selected from the US National *Malus* Collection (Geneva, NY, USA) based on fire blight resistance ratings available at Germplasm Resources Information Network (GRIN) (www.ars-grin.gov), 70 of which overlap with collection 1. Information regarding the accessions, including Plant Introduction (PI) number, name, species, origin, ploidy level and fire blight resistance ratings (available at GRIN) are reported in Supplementary Table S1.

The apple accessions from collection 2 were evaluated for fire blight resistance and susceptibility in a controlled greenhouse experiment. Three replicates of each accession were grafted via whip and tongue by interlocking corresponding cuts in the scion wood and ¼ inch ‘M.7’ rootstocks and potted in D40 large Deepots (Stuewe and Sons, Tangent, OR) in Sungrow #8/Fafard #2 (Sungrow Horticulture Agawam, MA) soil mix. The plants were grown in a greenhouse at a temperature between 21 and 24 °C with 12 h supplemental light and were watered and fertilized as needed.

### Genomic sequence and primers for *HIPM* and *DIPM* genes

The *HIPM* and *DIPM* gene sequences were detected in the ‘Golden Delicious’ double-haploid apple genome (GDDH13 v1.1)^43^ using a blast search with previously reported sequences^36,39^. The detected gene sequences were used as templates to design ~ 150 base pair amplicons to cover entire gene sequences using a custom python script (available at https://bitbucket.org/michelettd/ampseq-primer-design). The primers were designed using Primer3 (https://primer3.ut.ee, version 4.1.0) to amplify 150 ± 10 bp of fragments of the coding sequences from *HIPMs* and *DIPMs*. Besides the use of default parameters, all the primers were blast-searched against the GDDH13 v1.1^43^ reference genome to exclude putative repeat regions. The known SNPs were masked in the target regions to avoid annealing problems. On average, five primer pairs were selected for each gene in order to amplify gene fragments at intervals of approximately 300–600 base pairs via AmpSeq genotyping (see below) (Supplementary Table S2).

### Genomic DNA extraction and AmpSeq genotyping

Total genomic DNA from *Malus* accessions of Collection 2 (Supplementary Table S1) was extracted from young fresh leaves using the Wizard Genomic DNA Purification Kit (Promega, USA), according to the manufacturer's instructions. The concentration of extracted DNA was analyzed with a NanoDrop Spectrophotometer (Thermo Fisher Scientific, Gand Island, NY, USA) and the quality of DNA samples was assessed by running a 0.8% agarose gel at 80 V for 1.5 h. Genomic DNA was amplified with the previously designed and selected primer pairs (Supplementary Table S2) to amplify amplicon pools, which were sequenced on the Illumina MiSeq Platform (Illumina, San Diego, CA, USA), according to the AmpSeq protocol at the Genomics Platform Facility of Cornell University (Ithaca, NY, USA)^44^.

### Sequence analysis and variant detection

The sequences from AmpSeq run were demultiplexed using the barcode sequences. The raw sequences from 93 AmpSeq (Collection 2) were quality assessed using fastqc tool (https://www.bioinformatics.babraham.ac.uk/projects/fastqc/) and reads were trimmed and filtered to remove sequencing barcodes and low-quality bases using Trimmomatic software with the parameters: Trailing 20, Leading 20, SlidingWindow 4:15, AvgQual 20, MinLen 25^45^. For the remaining 145 accessions, whole-genome sequencing data were available, and the reads were filtered for quality as described above. The remaining high-quality reads were aligned against the GDDH13 v1.1^43^ genome with burrows-wheeler aligner (BWA) with default parameters. PCR duplicate reads were removed with Picard tools^43^.

Variants were identified using the HaplotypeCaller function of the Genome Analysis Toolkit (GATK version 4.1) using default parameters to detect SNPs and short Indels across the *HIPM* and *DIPM* gene sequences. The final SNP dataset was partitioned into three different datasets for (1) 145 *Malus* samples (Collections 1), (2) 93 AmpSeq samples (Collection 2), and (3) sum of the two collections. The variants were further filtered using a mean read depth threshold > 3. The variants were annotated using the coding and protein sequences of *HIPM* and *DIPM* genes from the GDDH13 v1.1^43^ genome. In addition, the gene annotations were obtained from the GDDH13 v1.1^43^ genome to annotate variants using the ANNOVAR program^46^.

### Assessment of genomic diversity

The genomic diversity across *HIPM* and *DIPM* gene sequences was determined by calculating nucleotide diversity (π) and fixation index (Fst) statistics across the three SNP datasets with the program VCFtools^47^. In addition, the population genetic structure was determined by performing principal component analysis (PCA) of the genetic variants with Tassel v5 software^48^.

### Fire blight inoculation and data collection

The fire blight inoculation of grafted plants was performed with highly virulent strains of *E. amylovora* (E2002a, Ea273, and E4001a)^49^ to evaluate varying levels of resistance and susceptibility. In 2018, the population was screened with a 1:1:1 ratio of E2002a, Ea273, and E4001a at a concentration of 1 × 10^9^ CFU/ml. In 2019, the only Ea273 strain was used at the same concentration as the prior year. The preparation of the inoculum consisted of plating the *E. amylovora* strain on King’s Medium B Base culture media, incubated at 27 °C for 2 days. Visible bacterial colonies were then scraped and added to 1× Phosphate Buffered Saline (PBS, pH 7.4) solution to halt growth of the bacteria. The bacterial suspension was quantified with a Smart Spec Plus Spectrophotometer (BioRad Hercules, CA) based on the optical density at 600 nm and diluted accordingly with 1× PBS to a concentration of 1 × 10^9^ CFU/ml. The inoculation was done by cutting the youngest leaf on the newest grown shoot with sterilized scissors dipped in the inoculum. During the early infection period, the temperature and humidity of the greenhouse was raised to 25–27 °C and 75–80%, respectively. After 12 days in 2018 and 8 days in 2019, the data of the fire blight infection was collected by measuring the leaf length (LL; cm) from the scissor cut to the end of leaf blade, leaf necrosis length (LN; cm) from the scissor cut to the visible lesion on the leaf, shoot length (SL; cm) of the current year’s growth from the node of the inoculated leaf and shoot necrosis length (SN; cm), the visible necrosis in the current year’s growth from the node of the inoculated leaf.

### Statistical analysis of fire blight data

Analysis of fire blight data was performed in R Version 3.6.2^50^. The datasets from each year were analyzed to remove any outliers. In 2019, plants with only old lignified leaves available for infection were noted and filtered out from further analysis. The remaining data were filtered for the genotypes with data for at least three replications. The leaf length (LL), leaf necrosis length (LN), shoot length (SL), and shoot necrosis length (SN), evaluated as above, were used to calculate percent leaf lesion length (PLLL), percent shoot lesion length (PSLL), and percent total distance (PTD) as follows;

$$ \begin{aligned} {\text{PLLL}} & = \left( {{\text{LN}}/{\text{LL}}} \right)*{1}00 \\ {\text{PSLL}} & = \left( {{\text{SN}}/{\text{SL}}} \right)*{1}00 \\ {\text{PTD}} & = \left( {\left( {{\text{LN}} + {\text{SN}}} \right)/\left( {{\text{LL}} + {\text{SL}}} \right)} \right)*{1}00 \\ \end{aligned} $$

PSLL is described as percent shoot necrosis (PSN) or percent lesion length (PLL) in previous literature^18,51^. For each genotype, the mean and standard deviation of all the replications were calculated for 2018 and 2019, separately. For the traits PLLL, PSLL, and PTD, a two-way analysis of variance (ANOVA) was performed to test for significant differences in phenotype across genotypes. To assess the homogeneity of the variances across the 2 years of data and disease traits within accessions, a non-parametric Fligner–Killeen test was conducted on the PLLL, PSLL, and PTD traits. In addition to the genotypic effects, the ANOVA model also accounted for the length of the inoculated leaf as covariates as well as the interaction effect between the individual genotype and the leaf length. The disease phenotype for each year was clustered to coerce accessions into classes of resistances using the partition k-means clustering approach. To perform clustering, the infection data for the traits PLLL and PTD of each genotype was scaled to a distribution mean of zero and a standard deviation of one via a Z-score normalization. Euclidean cluster distances were calculated using the ‘k-means’ package in R Version 3.6.2 based on the algorithm of Hartigan and Wong (1979). The number of clusters was determined using the ‘NbClust’ package in R Version 3.6.2 based on 30 unique indices that determine the optimal number of clusters representing a dataset^50,52^.

### Association between fire blight susceptibility and genomic variations

The fire blight phenotype data and the corresponding SNP genotypic data was used to conduct iterative marker-trait associations. The SNP genotype file was phased with Beagle v5.1^53^ and converted from the IUPAC ambiguity codes to a dominant genetic model, where ‘1’ represents the homozygous SNP states and ‘0’ represents the heterozygous sites. Each vector of genotype states for each marker was matched against the phenotype data for PLLL, PSLL, and PTD to perform iterative independent Kruskal–Wallis H-Tests with a custom loop created in R version 3.6.1^50^. The output provided *p* values for each SNP marker across all three traits and each year of data collection to identify significantly associated SNPs. Highly reliable marker-trait associations were determined through the criteria of high statistical significance with *p* < 0.001, their detection in multiple years and significance in leaf and shoot susceptibility. The fire blight susceptibility values of SNPs satisfying these criteria were plotted to observe the effect the genotype state has on specific traits. To find patterns across wild and domesticated *Malus* species, the proportion and frequency of species that possessed each selected SNP state was analyzed. The LD relationship of each pairwise marker combination was calculated in order to assess the relation of selected SNPs to the neighboring markers. This was performed first with the PLINK v1.90b6 genomic software to generate .ped and .info file format^54^. The files were imported in the Haploview v4.2 software to visualize marker relationships and find haploblocks^55^.

## Results

### Genetic diversity in homologs of *HIPM* and *DIPM*

Nine homologs of *HIPM* and *DIPM* genes were identified in the GDDH13 apple genome sequence^43^ with sequence length ranging from 1.4 to 3.7 kilobases (Table 1). These genes were present across 8 chromosomes in the apple genome. Two duplicated gene copies were present for *DIPM2, DIPM3*, *DIPM4*, and *HIPM1*, whereas *DIPM1* had a single copy present in the GDDH13 genome. It appeared that two copies of *DIPM2* located on Chr13 likely evolved through tandem duplication events due to their close proximity. The duplicated copies from remaining *HIPM* and *DIPM* genes were distributed across different chromosomes that might have occurred through whole-genome duplication events.

**Table 1:** Homologs of *DIPM* and *HIPM* genes, corresponding gene ids, chromosomes and their physical positions in Golden Delicious Double Haploid (GDDH13 v1.1)^43^ genome as well as number of SNPs (single nucleotide polymorphisms) and nucleotide diversity (π) across 93 *Malus* accessions.

Gene	Gene ID	Chromosome	Start	End	No. of variants	Nucleotide diversity (π)
*DIPM1*	*MD02G1176600*	*Chr02*	15,605,153	15,607,879	12	6.5 × 10^–03^
*DIPM2a*	*MD13G1036800*	*Chr13*	2,551,144	2,553,537	16	8.3 × 10^–03^
*DIPM2b*	*MD13G1036200*	*Chr13*	2,610,101	2,612,879	7	1.4 × 10^–02^
*DIPM3a*	*MD15G1078000*	*Chr15*	5,321,766	5,324,423	7	7.3 × 10^–03^
*DIPM3b*	*MDP0000303744*	*Chr08*	7,982,367	7,985,608	12	8.3 × 10^–03^
*DIPM4a*	*MD12G1097300*	*Chr12*	15,275,167	15,277,450	6	4.5 × 10^–03^
*DIPM4b*	*MD14G1094000*	*Chr14*	14,030,127	14,033,841	15	6.5 × 10^–03^
*HIPM1a*	*MD00G1089500*	*Chr00*	18,485,999	18,489,638	8	1.8 × 10^–02^
*HIPM1b*	*MD03G1224700*	*Chr03*	31,042,480	31,043,838	4	6.7 × 10^–03^

To assess the genetic diversity across *DIPM* and *HIPM* genes, we used publicly available whole-genome sequences from 145 diverse *Malus* accessions (collection 1). A total of 677 SNP variants were identified across these genes. The least variable gene was *DIPM4a* with 18 variants while the highest, 189 variants, were found in *HIPM1a* (Table 1). Nucleotide diversity (π) varied from 6.02 × 10^–03^ to 2.66 × 10^–03^ for *DIPM4a* and *DIPM2a*, respectively, with an average π of 1.38 × 10^–02^. The gene annotation analysis identified 65 total variants as nonsynonymous mutations that can lead to amino acid changes in a protein. There were also 3 frameshift and 2 non-frameshift Indels present in *HIPM1a*, *DIPM3a*, and *DIPM4b* genes. Furthermore, inference of population genetic structure using principal component analysis (PCA) with 677 SNP variants did not reveal any distinct clusters across the 145 *Malus* accessions (Supplementary Figure S5A,B). Altogether, these results demonstrated the high level of genomic diversity present in the *DIPM* and *HIPM* genes in apples.

The lack of phenotypic information for accessions in this dataset hindered the possibility of making an association analysis between SNP markers and fire blight susceptibility. Thus, amplicon sequencing was employed to amplify and assess the genetic diversity in the *HIPM* and *DIPM* genes in 93 selected apple accessions (collection 2; Supplementary Table S1)^44^. The latter analysis identified a total of 87 SNP variants across 9 *DIPM* and *HIPM* genes ranging from 4 (*HIPM1b*) to 16 (*DIPM2a*) (Table 1). The average π was 7.96 × 10^–03^ in 9 genes that varied from 4.5 × 10^03^ (*DIPM4a*) to 1.8 × 10^02^ (*HIPM1a*). Moreover, annotation of gene sequences revealed that 19 SNP mutations lead to changes in amino acid sequences of proteins in the *DIPM* gene homologs. At least one amino acid-changing mutation was identified in each of the *DIPM* genes. For instance, a single nucleotide change from “G” to “A” (position = 379) in *DIPM4a* can change the amino acid glycine to serine (Supplementary Table S2). Similarly, three different SNP mutations in the *DIPM4b* gene can change amino acid sequence from valine to aspartic acid, phenylalanine to leucine, and lysine to arginine. We further used the DIPM-HIPM SNP dataset to infer population genetic structure across 93 diverse *Malus* accessions from Collection 21 using PCA. Two accessions were removed from the analysis because they had more than 50% missing data. PCA identified three groups in the 93 *Malus* accessions. A set of 7 accessions including GMAL 2590, GMAL 2366, GMAL 1527.f1, GMAL 3052.g1, ‘Prince George’, and ‘Glabrata’ showed close grouping along PC1 values less than − 3.0 (Supplementary Figure S5B). Another set of 9 GMAL and “Kola'' accessions form a dispersed group with PC1 values between − 1.0 and − 2.0 (Supplementary Figure S5B). Finally, the third and largest group consisted of 77 accessions with highly diverse genetic backgrounds (Supplementary Figure S5B; Supplementary Table S1).

Comparative variant analysis between collection 1 and collection 2 indicated higher genomic variation and amino acid-changing mutations across *HIPM/DIPM* genes from 145 accessions, probably due to near complete coverage of these gene sequences in the GDDH13 apple genome. However, thirteen annotated variants were shared between 145 NCBI and 93 AmpSeq samples, out of which only 2 on the gene “MD08G1095100” were nonsynonymous (Table 1).

### Variation in fire blight susceptibility across diverse *Malus* accessions

Fire blight susceptibility in 93 *Malus* accessions was summarized as percentage of total infection as well as infection in the leaf and shoot (Supplementary Figure S2). The distributions of the leaf lengths and shoot lengths were normal across all accessions. The percent leaf lesion length (PLLL) was bimodal, centered around the extremes of the severity scale, the percent shoot lesion length (PSLL) was right skewed, and total lesion length measured as percent total distance (PTD) was also right skewed (Supplementary Figure S1). In 2018, the mean SL, LL, PSLL, PLLL, and PTD were 16.3 cm, 4.8 cm, 6.1%, 39.4%, and 12.1%, respectively. In 2019, the mean SL, LL, PSLL, PLLL, and PTD were 18.6 cm, 4.1 cm, 6.8%, 50.4%, and 13.7%, respectively. On the more resistant side of that range, there is a mix of accessions from *M. baccata*, and *M. fusca* as well as a few domesticated accessions such as ‘Koidu Rennett’. The highly susceptible accessions in the population included mostly *M. domestica* accessions such as ‘Idared’. The non-parametric Fligner-Killeen test for non-normal traits showed that the PLLL and PTD had homogenous variances across the accessions in the study (*p* value 2018/2019; PLLL: 0.8922, 0.1576, PTD: 0.1874, 0.5331). Overall, a wide range of responses to fire blight infection with varying levels of standard deviation were found across replicates for PLLL, PSLL, and PTD (Supplementary Figure S2A–C). Less susceptible accessions had relatively small standard deviations that increased as the values for PLLL and PTD increased consistently in both years (Supplementary Figure S2A,C).

The variation in PLLL, PSLL, and PTD during 2018 and 2019 was mainly determined by the genotypes (Table 2). For example, the ANOVA results showed that genotypic effects significantly influence fire blight susceptibility levels consistently for both leaf lesion (PLLL), shoot lesion (PSLL) and total lesion length in both years (Table 2). The k-mean clustering analysis of phenotypes showed larger ellipses for the susceptible class compared to the resistant class (Supplementary Figure S3). The clustering of genotypes based on the PLLL and PTD values identified three fire blight infection severity groups: susceptible, moderate, and resistant. The increase in fire blight severity of the susceptible group compared to the resistant group was 66.7% for PLLL and 27.2% for PTD in 2018. In 2019, the increase in fire blight severity of the susceptible group compared to the resistant group was 79.3% for PLLL and 36.3% for PTD.

**Table 2:** A summary of fire blight data collected in a controlled greenhouse during 2018 and 2019 for 93 *Malus* accessions.

Trait	2018		2019	
	Mean	*p* value	Mean	*p* value
Leaf length (cm)	4.8 ± 1.5	6.99e−05 ***	4.1 ± 1.6	8.14e−06***
Shoot length (cm)	16.3 ± 6.2	0.000164 ***	18.6 ± 7.8	1.52e−04 ***
Percent leaf lesion length (%)	39.4 ± 37.7	7.39e−05 ***	50.4 ± 40.9	< 2e−16***
Percent shoot lesion length (%)	6.1 ± 17.0	0.188	6.88 ± 17.5	0.0871
Percent total (disease) distance (%)	12.1 ± 18.3	0.0674	13.75 ± 18.7	1.69e−05***

The summarized data shows mean, standard deviation (SD), and analysis of variance (ANOVA) *p* value for fire blight susceptibility evaluations. Whereas *p* < 0.001: ***.

### Association between fire blight susceptibility and SNPs in *HIPM* and *DIPM*

**Figure 1:**
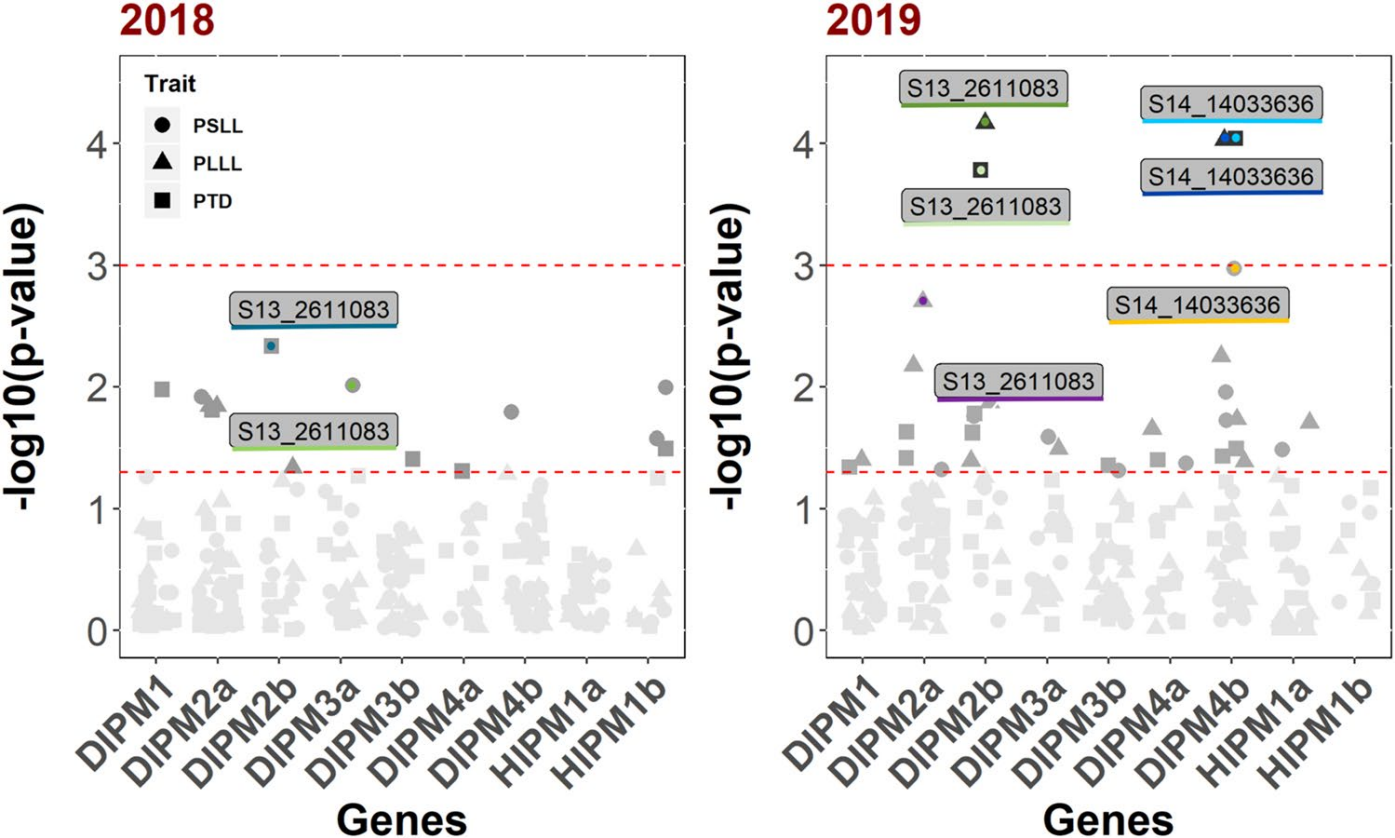
Manhattan plots showing SNP markers significantly associated with fire blight susceptibility traits in 93 *Malus* accessions. Candidate gene association analysis was done using Kruskal Wallis H-Test for fire blight susceptibility data collected during 2018 and 2019. The x-axis represents the gene IDs and corresponding markers on *HIPM*/*DIPM* genes. The y-axis shows the − log10 (*p* values) of marker-trait associations for fire blight severity traits; PSLL (percent shoot lesion length), PLLL (percent leaf lesion length), and PTD (percent total distance). The PSLL, PLLL, and PTD traits are displayed as a circle, triangle, and square, respectively. Two red lines parallel to the x-axis indicate varying levels of alpha significance (alpha = 0.05, bottom; alpha = 0.001, top). Increased value of the − log10 (*p* value) indicates a higher level of significance for the marker-trait association. The left and right panels are derived from data collected in 2018 and 2019, respectively. The color of dot in each symbol corresponds to the line under SNP label.

The Kruskal–Wallis H-test performed in R Version 3.6.2^50^ identified 30 unique SNP markers that showed significant association to fire blight susceptibility at *p* < 0.05, 2 of which showed high significance at *p* < 0.001 (Fig. 1; Supplementary Table S3). There are 6 SNPs out of 30 significant SNPs that result into non-synonymous amino acid changes in *HIPM* and *DIPM* gene homologs. In addition, SNP markers showing significant associations with PTD and PLLL were identified in both 2018 and 2019. A SNP marker, S13_2611083, showed a significant association (*p* < 0.05) with PLLL, PTD in 2018 and PSLL in 2019 (*p* value = 0.046, 0.005, 0.017). A high level of significance (*p* < 0.001) was found for PLLL and PTD in 2019 (*p* value = 6.76e−05,1.66e−04) (Supplementary Table S3). This marker was present in the *DIPM2b* gene on chromosome 13. Another SNP marker S14_14033636, on the *DIPM4b* gene region on chromosome 14, showed significant association (*p* < 0.001) with all three traits in 2019 (Fig. 1).

**Figure 2:**
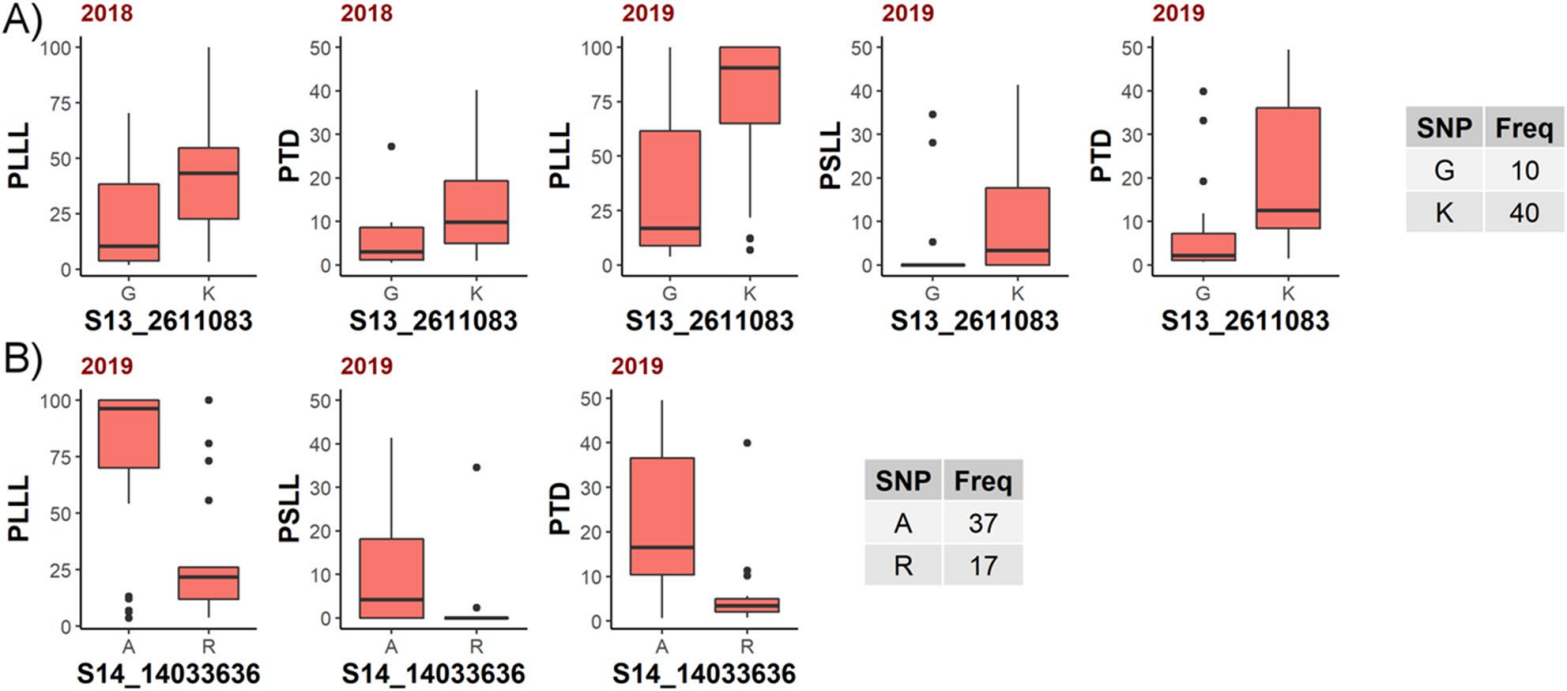
Boxplots of fire blight susceptibility at homozygous and heterozygous states of significantly associated SNP markers (**A**) S13_2611083 and (**B**) S14_14033636, respectively. The y-axis represents phenotypic values for percent leaf lesion length (PLLL), percent shoot lesion length (PSLL), and percent total distance (PTD). The “G” and “A” represent homozygous, and “K” and “R” represent heterozygous genotypic states for S13_2611083 and S14_14033636, respectively, on the x-axis. The table indicates allele frequency of homozygous and heterozygous SNP states across studied genotypes.

According to haplotype analysis, the SNPs significantly associated with fire blight susceptibility were in low linkage disequilibrium (LD) with flanking markers (Supplementary Figure S4A,B). Boxplots of the homozygous and heterozygous genotypes at these SNP states showed clear phenotypic differences in their susceptibility levels (Fig. 2). The heterozygous state for S13_2611083 SNP marker had higher rates of fire blight severity and conferred 1.8 and 2.6-fold increases in susceptibility in 2018 for PLLL, and PTD, respectively (Fig. 2A). The homozygous allele state of S14_14033636 SNP marker was associated with higher fire blight susceptibility and showed 5.4, 2.5, and 3.7-fold increases in susceptibility in 2019 for PSLL, PLLL, and PTD, respectively (Fig. 2B). In 2019 for the same marker, there were 2.5, 2.2, and 2.6 fold increases in susceptibility for PSLL, PLLL, and PTD from changing the homozygous SNP to the heterozygous.

**Figure 3:**
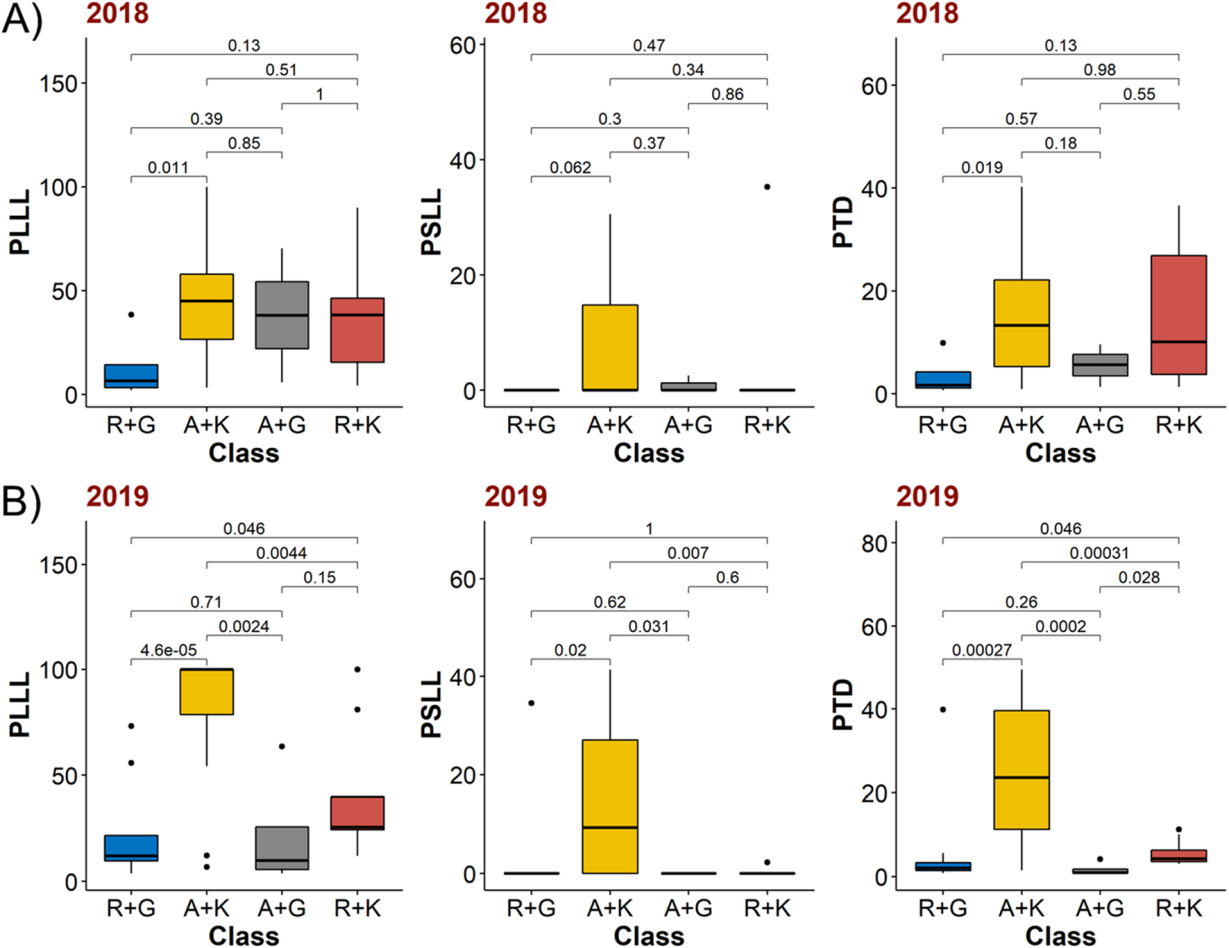
Pairwise Kruskal–Wallis H-tests to measure the additive effects of S13_2611083 and S14_14033636 significant SNP (single nucleotide polymorphism) markers in years (**A**) 2018, and (**B**) 2019. The x-axis represents one of the four possible combinations of homozygous and heterozygous genotype states from markers S13_2611083 (G or K) and S14_14033636 (A or R). The y-axis represents the fire blight susceptibility response in accessions carrying specific allele combinations. Fire blight susceptibility was measured as PLLL (percent leaf lesion length), PSLL (percent shoot lesion length), and PTD (percent total distance). The numbers on top of brackets for each subplot represent the *p* value of significance for pairwise comparison of each allelic combination for a specific trait and year.

We further observed additive allelic effects on the fire blight susceptibility from the most significant SNPs across *HIPM* and *DIPM* genes. For example, accessions possessing alleles linked with reduced susceptibility from both S13_2611083 and S14_14033636 markers showed an additive phenotypic response of significantly less fire blight susceptibility for PLLL (*p* value = 2018: 0.011, 2019: 0.0084) and PTD (*p* value = 2018: 0.019, 2019: 0.0011) and *vice-versa* (Fig. 3). The differences for PSLL were only significant in 2019 between the most susceptible class ‘A + K’ and the two less susceptible classes ‘R + K’, and ‘R + G’ (*p* value = 0.007,0.02; respectively) (Fig. 3).

**Figure 4:**
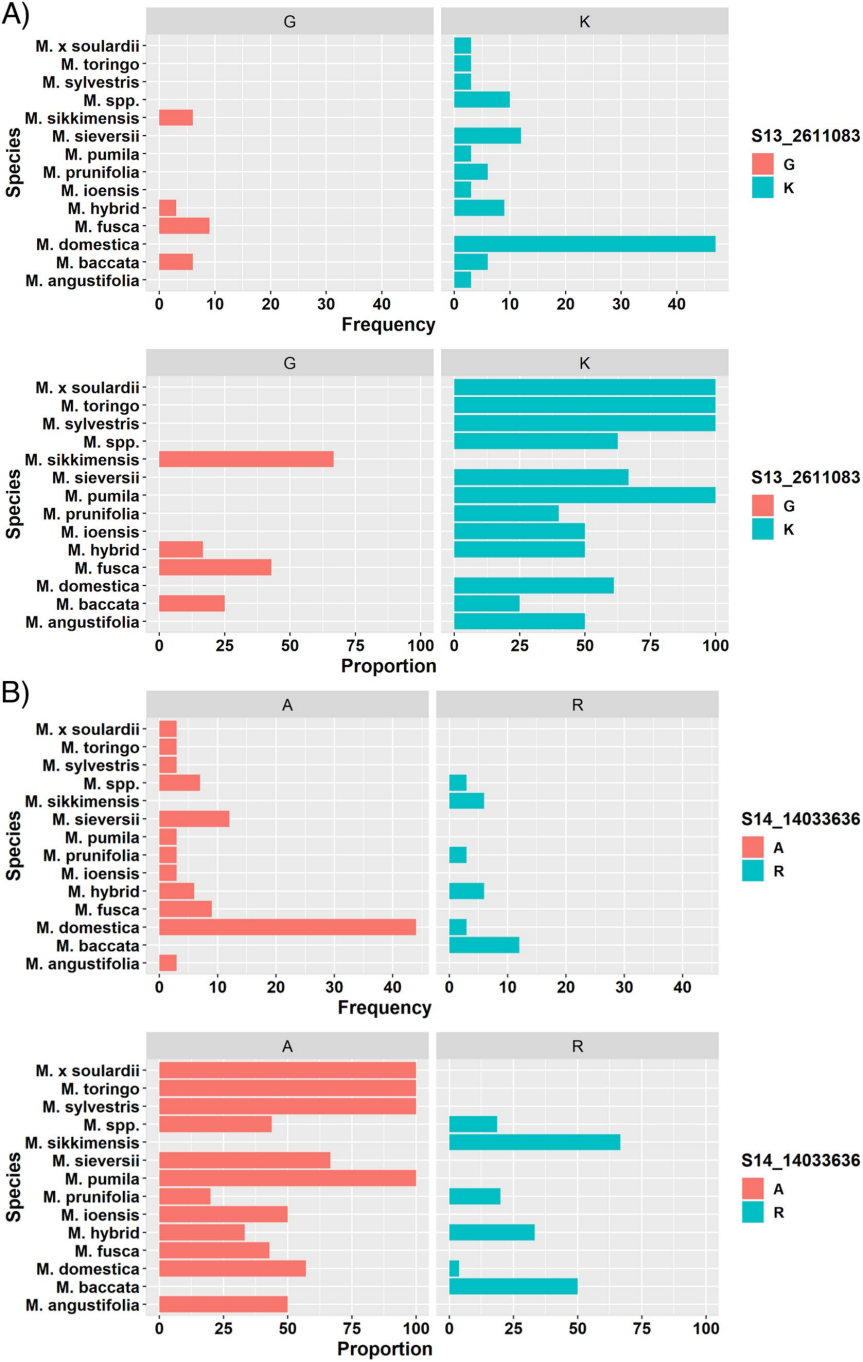
Horizontal barplots showing the frequency and proportion of *Malus* species that possess more fire blight susceptible heterozygous ‘K’ or ‘A’ or the less susceptible homozygous ‘G’ or ‘R’ SNP genotype at SNP marker (**A**) S13_2661083 and (**B**) S14_14033636, respectively. The y-axis indicates the 14 *Malus* species with either of the two SNP genotypes. The frequency is defined by the number of occurrences of a SNP across all the genotypes. The proportion is calculated by the frequency of a SNP divided by the total number of SNP, multiplied by 100.

The distribution of marker alleles across *Malus* species showed that the allelic states linked with higher susceptibility levels were mostly present in *M. domestica* accessions (Fig. 4A,B). In 2018, the average increase of susceptibility in domesticated accessions as opposed to wild accessions were 2.13, 6.12, and 5.22% for PLLL, PSLL, and PTD, respectively. In 2019, the average increases of susceptibility in domesticated accessions were 13.67, 6.68, and 5.5% for PLLL, PSLL, and PTD, respectively. SNP alleles with reduced levels of susceptibility mainly occurred in wild *Malus* species, whereas the proportion of higher susceptibility SNPs were found in *M. domestica* (Fig. 4A,B).

## Discussion

Genomic and genetic characterization of the S genes, which are responsible for successful pathogen infection, is a logical approach to achieve sustainable disease tolerance in improved cultivars^56,57^. Natural genetic variation in these genes can be tapped to develop cultivars with decreased fire blight susceptibility, a major goal of apple germplasm improvement efforts worldwide. The susceptibility genes, *HIPMs* and *DIPMs*, have been identified to regulate susceptibility and improved tolerance against fire blight in commercial apple cultivars^35,38,40,58^. We have found a great number of genomic variations in the 9 *HIPM* and *DIPM* genes across 145 diverse *Malus* accessions. We also found 30 SNPs with significant (*p* < *0.05*) associations to fire blight susceptibility, two of which had strong associations in two consecutive years of fire blight infection. A majority of the marker allelic states related to higher levels of susceptibility in these trait-associated markers were found in *M. domestica*, which is consistent with the current understanding that domesticated cultivars are generally susceptible to fire blight^7^. A higher proportion of species with the less susceptible allele were seen in wild species, with a shift to 50:50 in *Malus* hybrids. This is a possible indication of an inheritance pattern where a new mutation is introduced in a population and the more susceptible alleles are maintained and proliferated by selection during apple domestication. These susceptibility alleles were likely maintained in the domesticated germplasm due to fewer instances of cross-hybridization with wild apple species, to avoid unnecessary linkage drag of undesirable traits affecting fruit quality in apple cultivars.

The SNP marker S13_2611083 on *DIPM2b* showed significant association (*p* < *0.05*) with PLLL and PTD in 2018 and (*p* < *0.001*) in 2019. Robustness and stability of this SNP marker across two years makes it a reliable marker to screen for fire blight susceptibility and for use in marker-assisted breeding. Similarly, the strong association (*p* < *0.001*) of SNP marker S14_14033636 on the *DIPM4b* gene with PLLL and PTD indicates its potential use in marker-assisted selection to decrease fire blight susceptibility. The latter finding is consistent with previous work in which knocking out the *MdDIPM4* gene via CRISPR/Cas9 led to a 50% reduction in fire blight symptoms on notoriously susceptible ‘Gala’ and ‘Golden Delicious' cultivars^40^. These results also highlight utilization of candidate-gene based association studies to rapidly identify molecular markers linked to traits of interest for progeny and parent selection in breeding programs^59^. Breeding from a perspective of susceptibility rather than resistance can help resolve issues in tree fruit crops where linkage drag and cross incompatibility between *Malus* species and cultivated apples makes introgression of resistance alleles not feasible or very time consuming in many cases^11^. Hence, S-gene breeding opens the opportunity for breeders to more effectively improve fire blight resiliency of high value apple cultivars while not compromising on critical consumer focused traits.

Our results suggest that additive effects from multiple small effect loci in *HIPM* and *DIPM* genes contribute towards overall variation observed in disease susceptibility of domesticated apples. For example, combinations of positive alleles of S13_2611083 and S14_14033636 SNPs from two different loci have a stronger effect on fire blight susceptibility measures (i.e., PLLL and PTD). This is in contrast with the effect of major resistances contributed by wild apple germplasm, where a single gene or locus determined the fire blight resistance response without interacting with other loci in the genome^16,60–62^. The breakdown of single gene resistance/s by novel fire blight strains is highly likely, as shown for the breakdown of Robusta 5 (MR5) fire blight resistance by a deletion in the *avrRpt2* effector of Ea1189 *E. amylovora* strain^20^. Similarly, different strains of *E. amylovora* have been identified as virulent and avirulent on apple cultivars engineered for MR5 resistance^21^. These studies indicate that introgression of single gene resistances into commercial germplasm lie at a risk of breakdown by rapidly evolving pathogen strains. Emergence of a virulent strain for the particular resistance gene poses significant threat to breeding and selection efforts based on few major genes. Alternatively, additive alleles from different pathways can help achieve durable reduced susceptibility in a cultivar. For instance, selection of appropriate allelic combinations from HIPM/DIPM genes can lower successful pathogen infection, leading to reduced fire blight susceptibility. The root system can also influence fire blight susceptibility levels through co-regulated gene expression patterns and system-level interactions between carbohydrate and defense pathways^63^. Therefore, allelic combinations for the optimal root system can be used to breed rootstocks to further reduce fire blight susceptibility of grafted scions. These results depict a few examples related to disease susceptibility regulation, but apparently there are still several unexplored routes associated with disease susceptibility in the domesticated apples. Nonetheless, the presence of additive gene action from multiple pathways that are associated with disease susceptibility can make it harder for new pathogens to completely overcome the reduced disease susceptibility, which could be feasible to improve disease tolerance in apple breeding programs^56^.

The results presented in this study also represent strain-specific differences in host plant responses to fire blight as observed in the identification of different significant SNPs between 2018 and 2019. A mixture of three strains (Ea273, E4001a, E2002a) was used in 2018 while only E2002a was used in 2019 to inoculate the plants. The pure inoculum of the highly virulent E2002a strain generally showed higher disease incidence in 2019 for PLLL and PTD (Figure S2). In the mixed inoculum of 2018 infections, E2002a was present in lower concentrations and probably had more competition for host resources with Ea273 and E4001a. Furthermore, E2002a is a native North American strain, first discovered in Ontario, Canada off the “Jonathan” cultivar^64,65^. This strain likely had higher co-evolutionary compatibility with the North American domesticated accessions in this study. The chances of host–pathogen compatibility are mainly determined by the profile of effector proteins in individual *E. amylovora* strains that are responsible for fire blight pathogenicity and triggering of the hypersensitive response in *Malus*^6,66^. However, the strains used in this study exhibit high genome similarity^49^ and their considerably different phenotypic and genetic responses in host plants can lay out further investigations of *Malus-Erwinia* interactions.

In summary, a great genomic diversity was found in *HIPM* and *DIPM* genes in a wide set of *Malus* accessions and a candidate gene-based approach identified markers that were strongly associated with fire blight susceptibility. The validation of these markers could provide opportunities to use them in MAS to breed apple cultivars with reduced fire blight susceptibility. Moreover, characterization of S-genes can provide an alternative to improve fire blight resistance, either by breeding or genome-editing to avoid unnecessary risks associated with compromised fruit quality and breakdown of major resistance genes from wild *Malus* species.

## Supplementary information

Supplementary Figure S1.

Supplementary Figure S2.

Supplementary Figure S3.

Supplementary Figure S4.

Supplementary Figure S5.

Supplementary Table S1.

Supplementary Table S2.

Supplementary Table S3.
