# Author Correction: Candidate gene mapping identifies genomic variations in the fire blight susceptibility genes *HIPM* and *DIPM* across the *Malus* germplasm

**DOI:** 10.1038/s41598-021-82641-2

**Published:** 2021-01-27

**Authors:** Richard Tegtmeier, Valerio Pompili, Jugpreet Singh, Diego Micheletti, Katchen Julliany Pereira Silva, Mickael Malnoy, Awais Khan

**Affiliations:** 1 Plant Pathology and Plant‑Microbe Biology Section, Cornell University, Geneva, NY 14456 USA; 2 Research and Innovation Centre, Fondazione Edmund Mach, San Michele all’Adige, Italy

Correction to: *Scientific Reports* 10.1038/s41598-020-73284-w, published online 01 October 2020

This Article contains a typographical error in the Materials and methods section under subheading ‘Association between fire blight susceptibility and genomic variations’.

“The SNP genotype file was phased with Beagle v5.1^53^ and converted from the IUPAC ambiguity codes to a dominant genetic model, where ‘1’ represents the homozygous SNP states and ‘0’ represents the heterozygous sites.”

should read:

“The SNP genotype file was phased with Beagle v5.1^53^ and converted from the IUPAC ambiguity codes to an additive genetic model, where ‘1’ represents the homozygous SNP states and ‘0’ represents the heterozygous states.”

