# Supplementary figures and images for: Candidate gene mapping identifies genomic variations in the fire blight susceptibility genes HIPM and DIPM across the Malus germplasm
Source: Sci Rep. 2020 Oct 1;10:16317. doi: 10.1038/s41598-020-73284-w (PMC7529791; doi:10.1038/s41598-020-73284-w)

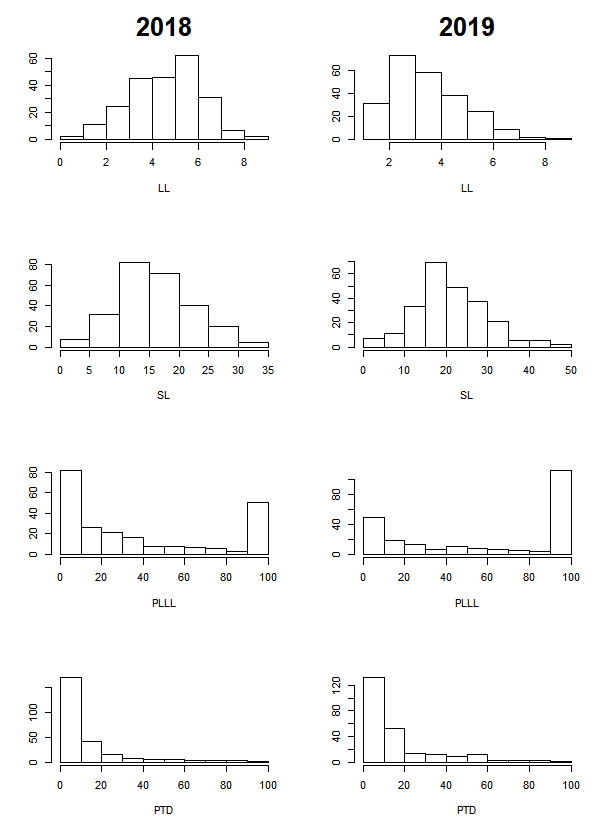

Supplement: Supplementary file 1 — Supplementary Figure S1. [file 41598_2020_73284_MOESM1_ESM.tiff]

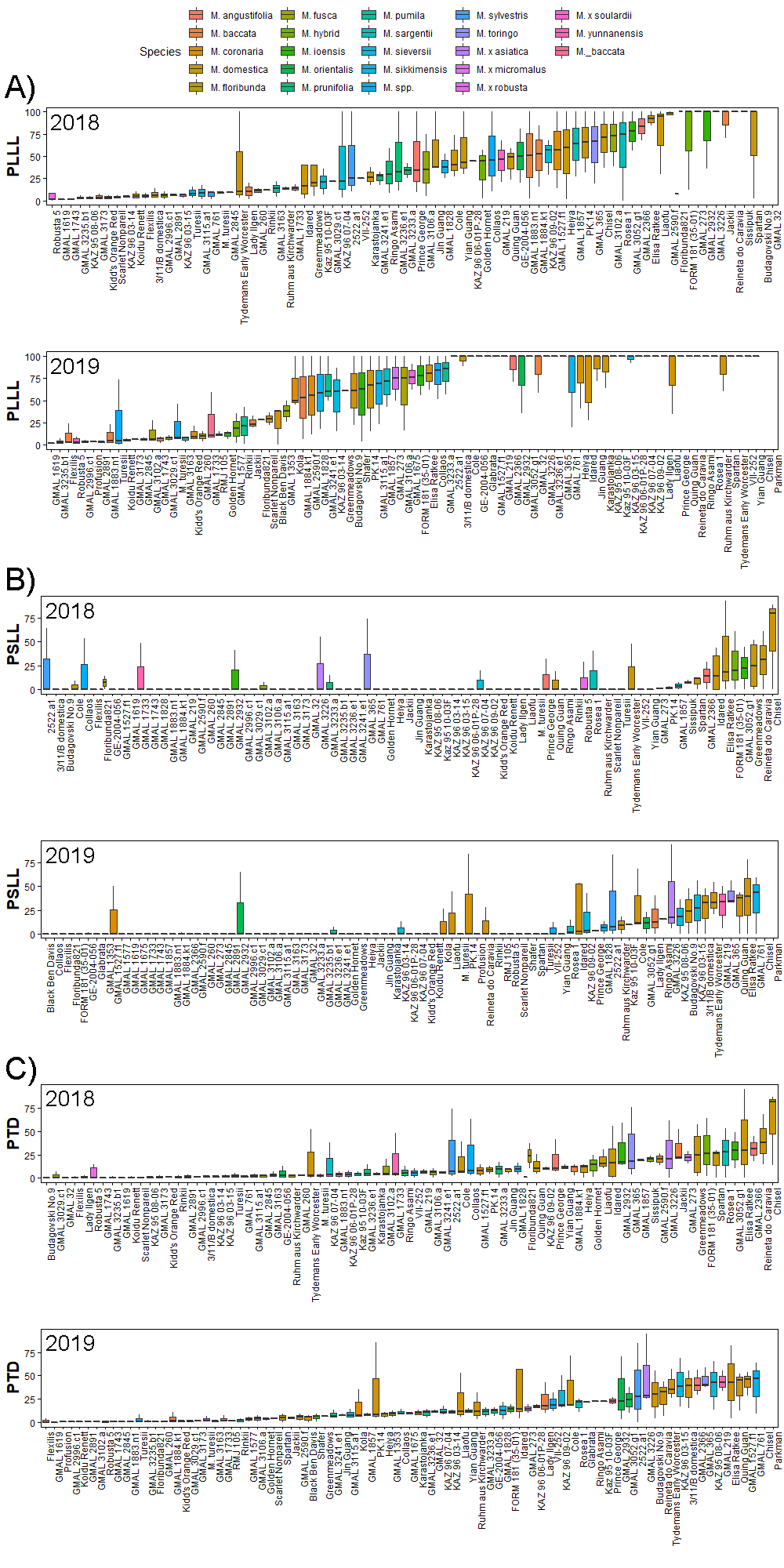

Supplement: Supplementary file 2 — Supplementary Figure S2. [file 41598_2020_73284_MOESM2_ESM.tiff]

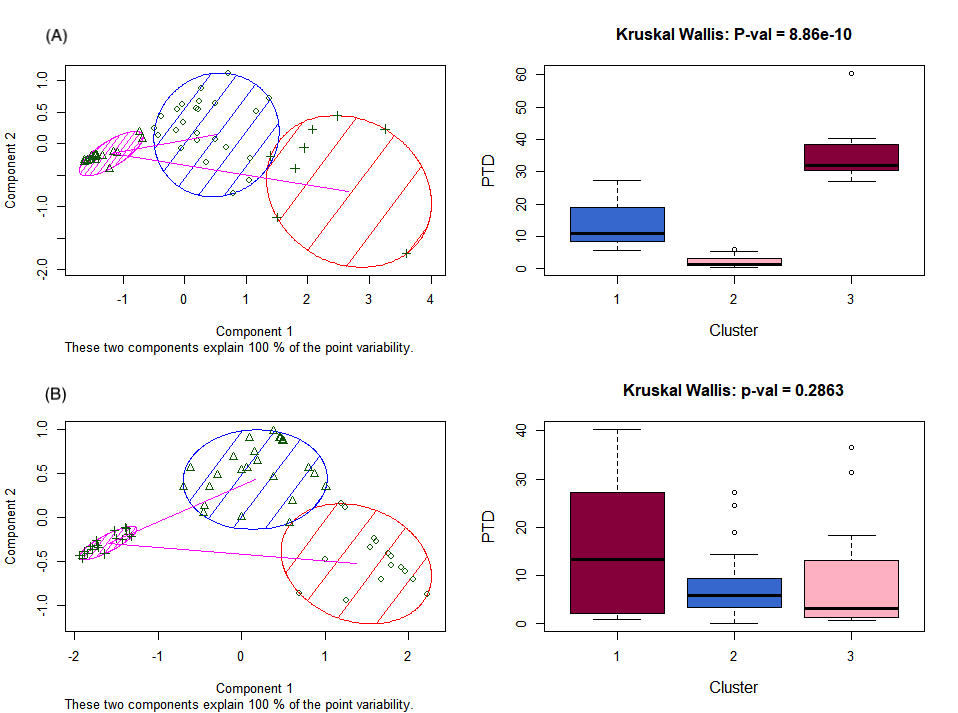

Supplement: Supplementary file 3 — Supplementary Figure S3. [file 41598_2020_73284_MOESM3_ESM.tiff]

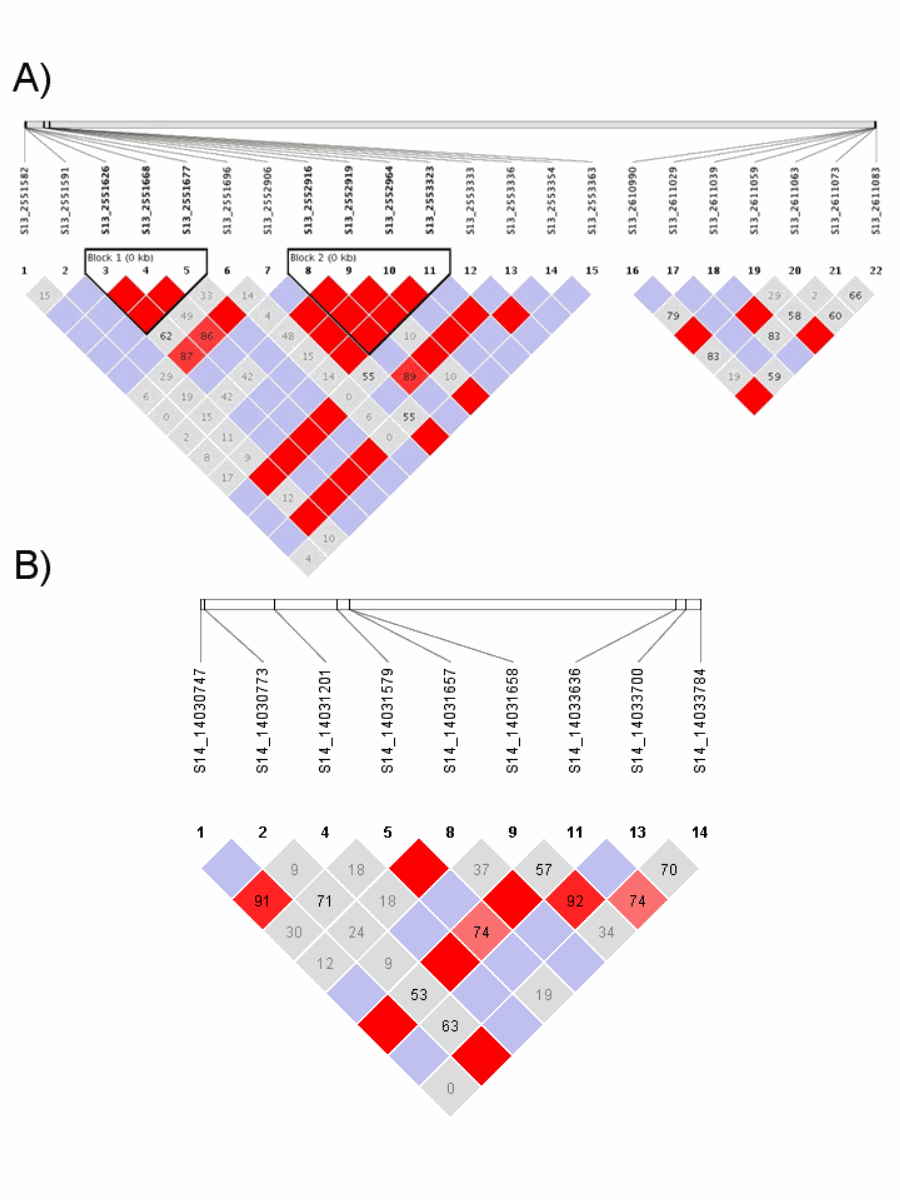

Supplement: Supplementary file 4 — Supplementary Figure S4. [file 41598_2020_73284_MOESM4_ESM.tiff]

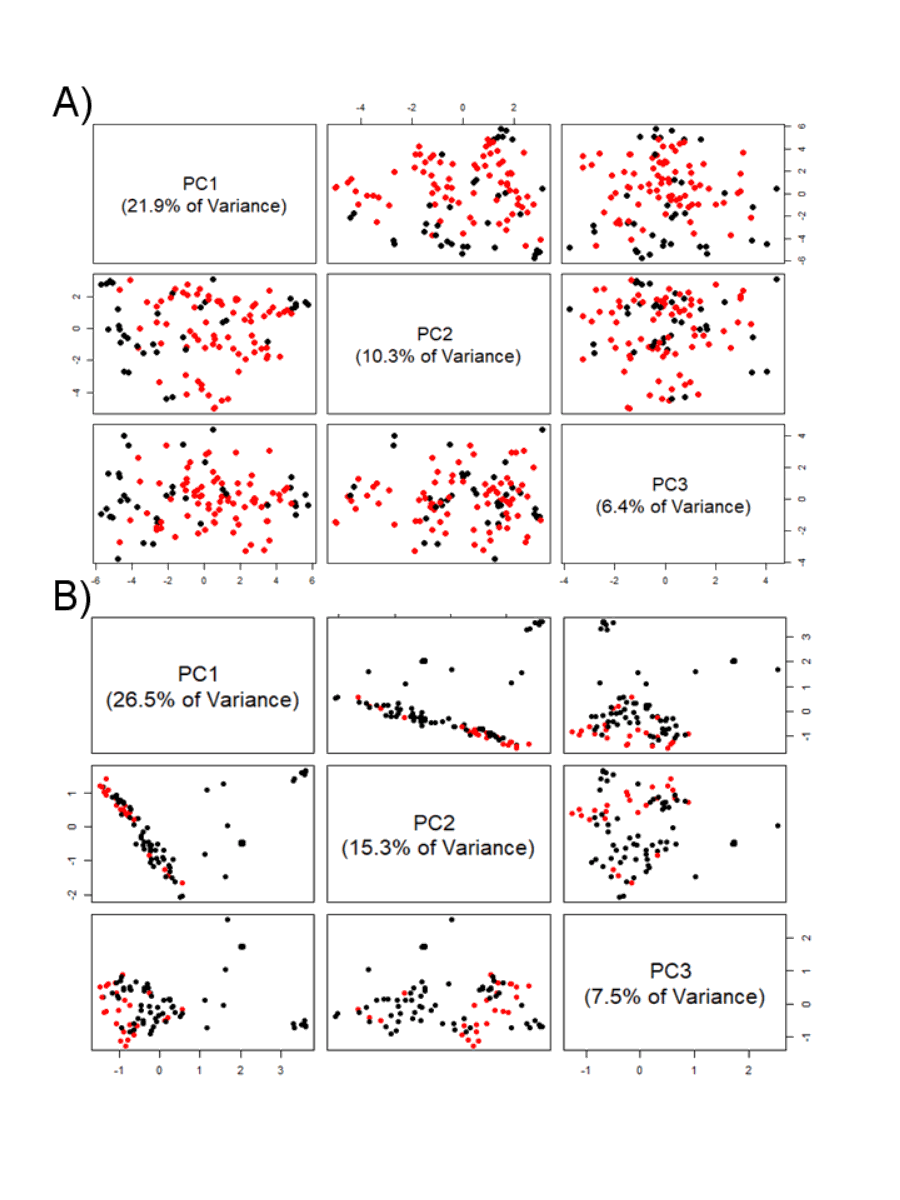

Supplement: Supplementary file 5 — Supplementary Figure S5. [file 41598_2020_73284_MOESM5_ESM.tiff]
